# A novel nonlinear dimension reduction approach to infer population structure for low-coverage sequencing data

**DOI:** 10.1186/s12859-021-04265-7

**Published:** 2021-06-26

**Authors:** Miao Zhang, Yiwen Liu, Hua Zhou, Joseph Watkins, Jin Zhou

**Affiliations:** 1 Department of Epidemiology and Biostatistics, University of Arizona, 1295 N. Martin Ave., 85724 Tucson, USA; 2 Department of Mathematics, University of Arizona, 617 N. Santa Rita Ave., 85721 Tucson, USA; 3 Interdisciplinary Program in Statistics and Data Science, University of Arizona, 617 N. Santa Rita Ave., 85721 Tucson, USA; 4 Department of Biostatistics, University of California, Los Angeles, 650 Charles E. Young Dr. South, 90095 Los Angeles, USA; 5 Department of Medicine, UCLA David Geffen School of Medicine, Los Angeles, CA USA

**Keywords:** Dimension reduction, Non-linear kernel, Low-coverage, Population structure, Data-adaptive

## Abstract

**Background:**

Low-depth sequencing allows researchers to increase sample size at the expense of lower accuracy. To incorporate uncertainties while maintaining statistical power, we introduce MCPCA_PopGen to analyze population structure of low-depth sequencing data.

**Results:**

The method optimizes the choice of nonlinear transformations of dosages to maximize the Ky Fan norm of the covariance matrix. The transformation incorporates the uncertainty in calling between heterozygotes and the common homozygotes for loci having a rare allele and is more linear when both variants are common.

**Conclusions:**

We apply MCPCA_PopGen to samples from two indigenous Siberian populations and reveal hidden population structure accurately using only a single chromosome. The MCPCA_PopGen package is available on https://github.com/yiwenstat/MCPCA_PopGen.

**Supplementary Information:**

The online version supplementary material available at 10.1186/s12859-021-04265-7.

## Background

High-throughput sequencing technologies are capable of generating billions of short sequence reads on scale [6]. Different sequencing designs and platforms provide options balancing accuracy and cost. High-depth whole-genome sequencing identifies nearly all variants along the genome with high confidence but at high cost [3, 4, 14]. As a cost-effective alternative, low to medium depth next-generation sequencing (NGS) has lower accuracy, especially for rare-variant identification and genotype calling, but at much lower cost  [2, 23, 30, 40, 42]. Low coverage sequencing technology ($<5$x) has shown to be valuable in a variety of population genetic issues, e.g, in population structure [37], in conservation biology [12], in ancient DNA [1], and in single-cell RNA sequencing [15]. In humans, ultra low-sequencing technology has been widely adopted for non-invasive prenatal tests of the maternal plasma [24]. Compared with high-coverage sequencing data, genotypes from low-coverage sequencing data are noisier and thus bring higher levels of uncertainty [29]. Downstream analyses based on the raw sequencing data incorporating uncertainties are advantageous and comparable to high-depth NGS [14, 22]. Therefore, researchers can afford to sequence more samples at comparable cost with minimal sacrifice in statistical power.

One fundamental dimension reduction technique for NGS data is principal component analysis (PCA) [19]. This analysis determines the principal components (PCs), i.e., the linear projection of the original variables onto a low dimensional vector space that maximally explains the variance of the data. Among its many applications, PCA is a widely adopted tool in genetic studies to infer population structure [26, 27, 32, 33, 44]. However, PCA is not designed to reveal the nonlinear relationship that may arise, for example, from the uncertainties in low-depth genomic data. Several methods, including IsoMap [41], locally linear embedding (LLE) [36], and Kernel PCA (KPCA) [39] have been developed to capture nonlinear patterns. KPCA enables us to construct nonlinear versions of the PCA algorithm and has been successfully applied to gene expression data for the classification of samples [25, 35]. However, KPCA suffers from two major limitations: 1) the kernel must be pre-specified; 2) the corresponding transformation is identical at each locus. However, the form of transformation may depend upon the alleles’ characteristics, e.g., rare or common alleles (see Additional file 1: Fig. S1).

**Fig. 1:**
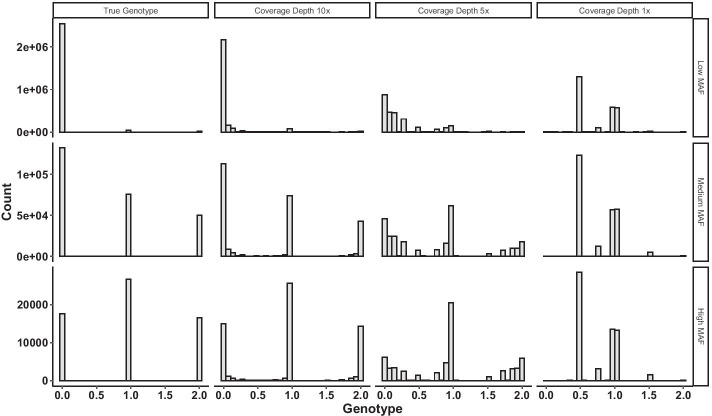
The histograms of true genotypes across all 19,530 SNPs and the histograms of genotype dosage values for coverage depth 10, 5, and 1 when MAFs are low ($\hbox {MAF}<0.1$), medium ($0.1\le \hbox {MAF}<0.4$), and high ($\hbox {MAF}\ge 0.4$). Genotypes dosage are the posterior mean of the genotype under additive coding. With values 0, 1 and 2 assigned to the genotypes (major, major), (major, minor) and (minor, minor), respectively, the genotype dosage, ${\text{ DS }} = \Pr (1 |{\text{ Data}})$ + 2$\Pr (2 | {\text{ Data}})$, where $\Pr (1 |{\text{ Data}})$ and $\Pr (2 |{\text{ Data}})$ denote the conditional (“posterior”) probabilities for the genotypes (major, minor) and (minor, minor)

To optimize the usage of ultra low-coverage sequencing datasets, we propose an extension of a data-adaptive approach, Maximally Correlated Principal Component Analysis (MCPCA) [11], which naturally addresses the first two limitations. To address the third, our method uses genotype likelihoods rather than any single genotype. Taking into account the uncertainty of raw sequencing reads provides an opportunity to model the nonlinear patterns in population genetics data. In particular, we employ a continuous value, i.e, dosage (see Fig. 1), to summarize the uncertainty in genotype calling. MCPCA is designed to determine a transformed dosage value, $x_j\mapsto \phi _j(x_j)$, at each locus *j* to maximize the sum of a pre-specified number of eigenvalues of the transformed dosage covariance matrix (the Ky Fan norm [10]). We name our method MCPCA_PopGen, aiming to analyze the population structure for low-coverage sequencing data. It applies MCPCA to genotype dosages and finds the optimal transformations to explain a maximum proportion of the variances in the data. Our simulation reveals two major properties of MCPCA_PopGen for analyzing low-coverage sequencing data. For a locus with a low minor allele frequency (MAF), the transformation emphasizes the uncertainty in calling between heterozygous and the major homozygous loci. On the other hand, the transformation is more linear when variants are common (see Additional file 1: Fig. S1). We performed extensive simulations and demonstrated the benefit of MCPCA over standard PCA and KPCA for low-coverage data. We applied MCPCA to two indigenous Siberian populations. The optimized MCPCA explains a much higher percentage of the variance and more clearly distinguishes these two populations even when limited to the genetic information from a single chromosome.

## Results

### Simulation studies

#### Variance explained by MCPCA_PopGen

We evaluate the MCPCA method (MCPCA_PopGen) using three types of genotype callings, (1) true genotypes, (2) observed genotypes with errors, and (3) genotype dosage. Genotypes were simulated using *ms* package [17] from three populations (African, Caucasian, and Asian) (*ms* commands to simulated genotypes were included in the Additional file 1: Sect. 3). They took value from $\{0,1,2\}$, representing the minor allele counts carried by each individual at each locus. Observed genotypes were generated by perturbing the known genotype under specified coverage depths as developed in  [8]. Genotype dosage is the posterior mean of the genotype calls under additive coding (Fig. 1) [43]. Details of the simulation procedures are provided in the “Methods” section. As illustrated in Table 1, observed genotypes with coverage depth below $10\times$ have high error rates in these simulated datasets. When the coverage depth is low, the “best-guess” genotypes frequently differ from the true genotypes. In our simulation studies, we evaluate the total variance explained by the top *q* MCPCs. We compare the computational efficiency across different *q* and different number of Single nucleotide polymorphisms (SNPs) used to generate PCs. Finally we compare the performance of MCPCA_PopGen with PCA and KPCA.

**Table 1:** The percentage of error calling and the average Phred quality scores for observed genotypes across all 19530 SNPs in simulated datasets

Coverage depth	Percentage of error calling (%)	Mean quality score (SD)
$1\times$	70.49	3.37 (1.34)
$5\times$	12.59	15.28 (7.45)
$10\times$	3.19	29.53 (13.27)

**Fig. 2:**
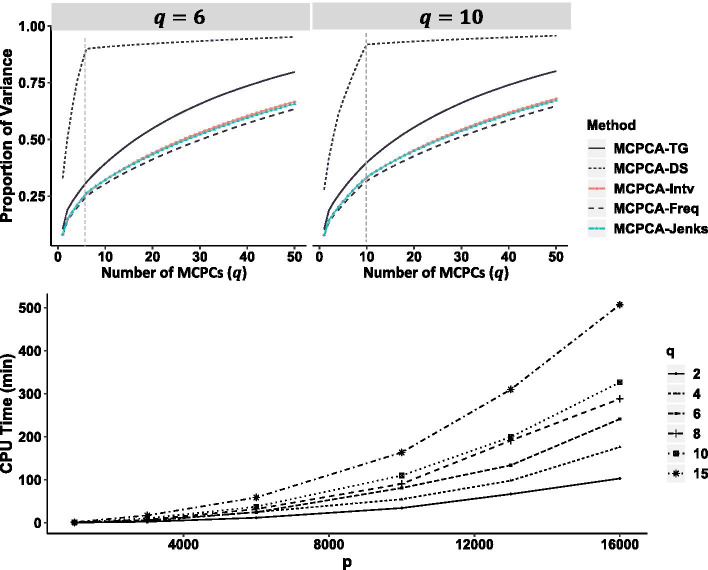
(top) The proportions of the variances explained by the first 6 and 10 PCs. The data consists of 150 samples, each having 1458 SNPs. (bottom) The CPU time of implementing MCPCA_PopGen given different choice of *q* when the number of SNPs ranges from 1000 to 16,000. MCPCA-TG: MCPCA applied to the true genotype data; MCPCA-DS: MCPCA applied to the genotype dosage data; MCPCA-Intv, MCPCA-Freq, and MCPCA-Jenks: MCPCA using the equal width, equal frequency, and Jenks binning methods

Determine the optimal number of MCPCs Choosing the number of maximally correlated principal components *q* is essential. A small *q* may result in loss of information. The computational time increases if a large value of *q* is selected. To provide a practical guide in choosing the number of MCPCs, we demonstrate in Fig. 2 how much more of the variances is explained with increasing values of *q*. The MCPCA algorithm is applied to the true genotype data (MCPCA-TG), dosage data (MCPCA-DS), and discretized dosage data given $q=6$ and $q=10$ respectively, i.e., MCPCA-Intv, MCPCA-Freq, and MCPCA-Jenks represent MCPCA algorithm applied to discretized dosage data with different binning methods : equal width, equal frequency, and Jenks binning). Please refer to “Methods” section for details.

We used the proportion of variance explained by the true genotype (MCPCA-TG) as a baseline. As showed in Fig. 2 (top panel), MCPCA-DS explains a much larger proportion of variances than MCPCA-TG, indicating overfitting due to the over-determined number of categories. By implementing MCPCA on an optimally discretized dosage values (MCPCA-Intv, MCPCA-Freq, and MCPCA-Jenks), we avoid overfitting. Note that all three discretization methods achieve comparable proportions of explained variances to that of MCPCA-TG. We also illustrate how the CPU time for implementing the proposed algorithm changes as we vary *q* and the number of SNPs *p* in the data. Given that *q* ranges from 2 to 15, the CPU time has a polynomial growth as *p* increases. The computational complexity of MCPCA algorithm for each iteration is $O(p^3+np^2)$ [11]. For $n\gg p$, the algorithm is nearly linear with *n*, which makes this approach suitable for data sets with a large number of individuals (e.g., biobank scale studies). When the number of SNPs *p* substantially exceeds the sample size *n* or when they are in the same scale, the MCPCA_PopGen algorithm runs in cubic time $O(p^3)$. To balance the interpretability, effectiveness, and efficiency of our algorithm, we suggest a choice of *q* at most 20 when *p* is large, and a pruning procedure for choosing SNPs for analysis should also be adopted [5]. Our analysis were performed using 11 cores and 6 GB memory computing resources.

**Fig. 3:**
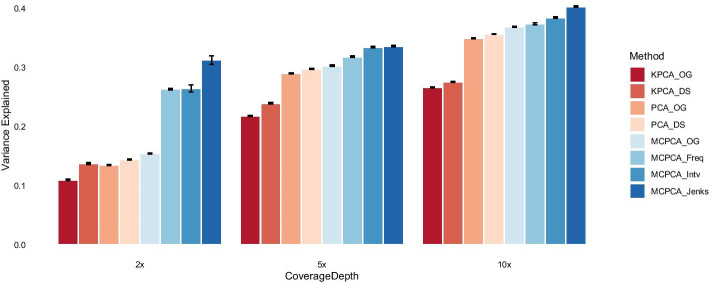
The average of variances explained by the top 10 PCs over 100 simulation replicates. In these scenarios, KPCA-TG, PCA-TG, and MCPCA-TG explained 0.2893, 0.3768, and 0.3982 of the totoal variances, respectively. Error bars represent one standard deviation away from the mean

Performance comparisons The performance of MCPCA_PopGen was compared with that of PCA with respect to the proportion of variances explained by the first *q* PCs. The results were summarized over 100 simulation replicates. In all scenarios, we set $q=10$. Figure 3 displays the barplot of variances explained by the top 10 PCs over 100 simulation runs. In all scenarios, MCPCA or PCA on dosage data show better performance than that on the observed genotypes (PCA-OG and MCPCA-OG), indicating that dosage values preserve more information by taking into account the uncertainty in genotype calling. MCPCA outperforms PCA under different discretization methods in all scenarios, especially when the coverage depth is low (Fig. 3, left panel). As illustrated in Additional file 1: Fig. S1, MCPCA finds nonlinear transformations of dosage values with low MAF, emphasizing the uncertainty in calling between heterozygous and the major homozygous loci. Among the three discretization methods, MCPCA using the Jenks discretization has the highest explained variance. We have also applied KPCA to the observed genotypes (KPCA-OG) and dosage genotypes data (KPCA-DS). Instead of Gaussian kernel, the polynomial kernel was adopted since KPCA had better performances with a polynomial kernel in our simulation studies. In all scenarios, KPCA did not perform well when coverages were $>5$x. When coverage was low (i.e., 1x), it has a similar performance as PCA. These results suggest that an adaptive transformation according to data coverage depth is needed rather than the “one-size-fits-all” approach.

#### Prediction accuracy of MCPCA

**Fig. 4:**
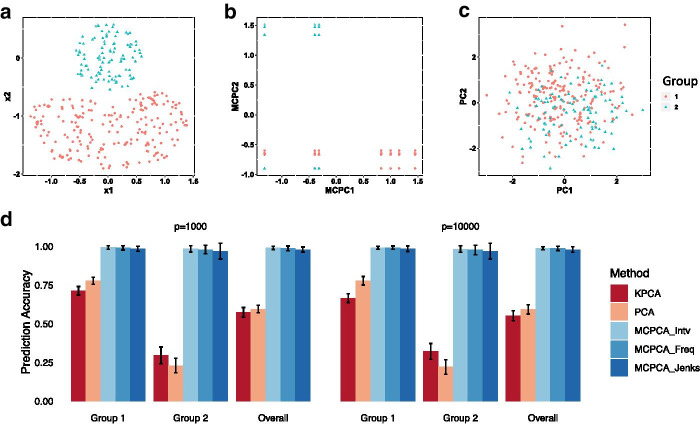
**a** Illustrates that two groups of samples are generated such that a nonlinear curve of $x_1$ and $x_2$ separates the two groups. **b** and **c** Present the visualization of the two groups using the first two components from MCPCA and PCA, respectively. **d** Shows the barplots of prediction accuracy of MCPCA, PCA, and KPCA under various scenarios. Error bars represent one standard deviation away from the mean

In this section, we illustrate the performance of the MCPCA method in predicting sample identities by utilizing nonlinear patterns among predictors. The true model is demonstrated in Fig. 4a. Two groups of samples were simulated in a way such that a nonlinear curve of $x_1$ and $x_2$ may give a clear separation of the two groups (Fig. 4a). We further generated $p-2$ predictors from a standard normal distribution, where $p=1000$ and $p=10000$. The sample sizes for group 1 and 2 were set to be 200 and 100, respectively. We applied MCPCA, PCA, and KPCA to the simulated data and projected the samples into the two-dimensional spaces formed by their embeddings. MCPCA distinguished the two groups more clearly (Fig. 4b and c). To evaluate the prediction accuracy, we trained random forests to predict sample identities using the two-dimensional embeddings generated by MCPCA, PCA, and KPCA. When implementing MCPCA, three discretization methods (MCPCA-Freq, MCPCA-Intv, and MCPCA-Jenks) were used (see “Methods” section). The within-group and overall accuracy of the predictions were measured through out-of-bag (OOB) prediction errors over 100 simulation replicates. In all scenarios, MCPCA with different discretization methods achieved higher accuracies than PCA and KPCA and were robust in both groups, even when *p* was much larger than the sample size (Fig. 4d). To summarize, the MCPCA method enables the discovery of nonlinear transformations of predictors, whose linear combinations provide a better prediction accuracy.

### Application to Siberian population

Based on a low-coverage whole exome sequencing data, [16] reported the evidence for cold adaptation in two indigenous Siberian populations, the Nganasan (nomadic hunters, NGA, $n=21$, $\sim 6\times$ coverage) from the Taymyr Peninsula in the Arctic Ocean, and the Yakut (herders, YAK, $n=21$, $\sim 4\times$ coverage) of North-Central Siberia (More detail of the data is provided in [16]). This low-coverage data set provides an excellent opportunity to test the ability of MCPCA_PopGen to classify the two groups. Utilizing genotype posterior probabilities extracted from Binary Sequence Alignment/Map format (BAM) files by the software ANGSD [22], we calculated the dosage values. For comparison, we also applied ngsPopGen [13] and PCA (PCA-DS) to these data. Like MCPCA_PopGen, the approach in ngsPopGen approximates the covariance matrix among individuals using posterior probabilities of sample allele frequencies, thus accounts for the uncertainty of low quality and/or coverage sequencing data. While for PCA-DS method, instead of using posterior probabilities, we calculated the covariance matrix using genotype dosage. As the posterior mean of the genotype, dosage also summarizes the uncertainty in genotype calling. Eigen-decomposition of the two resulting covariance matrices then enables us to perform PCA.

**Fig. 5:**
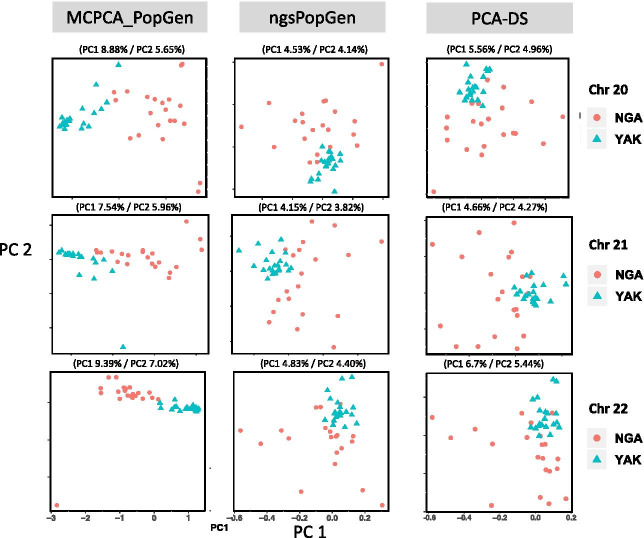
(left) MCPCA plot for chromosome 20–22; (middle) PCA plot (ngsPopGen) for chromosome 20–22; (right) PCA plot (PCA-DS) for chromosome 20–22 for the Nganasan (NGA) and Yakuts (YAK) samples

**Fig. 6:**
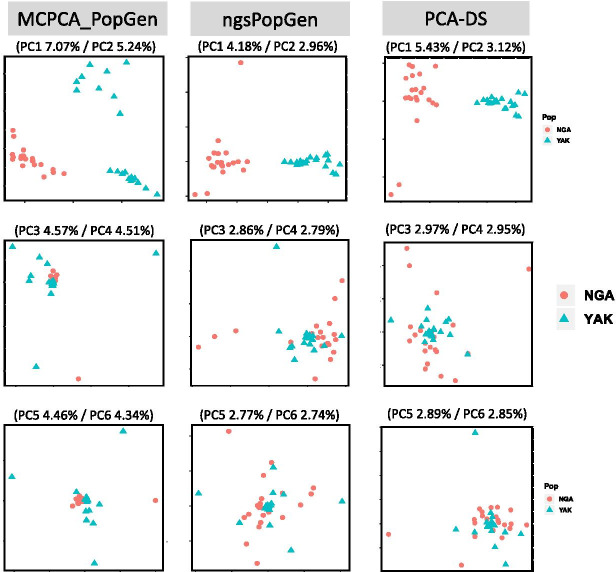
(left) MCPCA plot for top 6 PCs; (middle) PCA plot (ngsPopGen) for top 6 PCs; (right) PCA plot (PCA-DS) for top 6 PCs. 51,673 SNPs across Chromosome 1–22 were used

We illustrated the performance of MCPCA_PopGen using Figs. 5 and 6. For Fig. 5, we set $q=20$ and applied MCPCA_PopGen, ngsPopGen, and PCA-DS to the data obtained from chromosomes 20, 21, and 22. First note that MCPCA_PopGen more clearly separates the two populations. In addition, the first two principal components of MCPCA_PopGen explain at least 13% of the variance, whereas ngsPopGen and PCA-DS explain around 8% - 10%. In preparation for Fig. 6, we called posterior probabilities of the genotype likelihood across all 22 human chromosomes. After filtering, this provides a total of 51, 673 SNPs for analysis. We display the top 6 PCs from MCPCA_PopGen, ngsPopGen, and PCA-DS. The MCPCA plots are consistent with reported histories of these two groups. As shown in [34], the Yakuts are more admixed (with Mongolian populations) than the Nganasan. The top plot seems to show two somewhat distinct Yakuts populations. The data were taken from two villages which do not match the clustering in the MCPCA plot [16]. However, analysis of ancient DNA [21] reveals evidence of Yakuts parent-child relationships in graves 70 km apart, indicative of a mobile population. As noted in [28], PCA may not be able to distinguish between migration and a population split. Both [20, 34] found evidence of severe bottlenecks in the Nganasan. This is displayed in the plot showing that except for one individual, the MCPCA plots for the Nganasan in both the PC3/PC4 and PC5/PC6 plots are very tightly clustered.

## Discussion

In genetic studies, PCA is a widely adopted dimension reduction tool to infer population structure and to adjust for population stratification. Unlike high-density SNP arrays, new sequencing technologies allow us to model the genotype uncertainty of raw sequencing reads rather than make a hard decision of any single genotype and to provide options balancing between accuracy and cost. New approaches are needed in order to make effective use of this type of data better.

In this article, we introduce a dimension reduction approach for low-coverage sequencing data. To account for the genotype uncertainty, we propose the use of dosage values instead of the discrete genotypes. By considering both the genotype uncertainty and nonlinear correlations, our method transforms each SNP sequentially by maximizing the sum of top *q* eigenvalues of the transformed covariance matrix. The advantage of our method is that the data are used to optimize the transformation for each SNP, an approach that is not permitted in KPCA. For our simulations, we learned that the transformation is more nonlinear, emphasizing the difference between heterozygous and the major homozygous genotypes, for the SNPs with low MAF and more linear for common variants. To balance among computational feasibility, issues with overfitting, and statistical power, we analyzed three candidate methods to discretize dosage values. In simulation studies, we demonstrate that our method achieves higher fractions of the variance explained by meta-features when compared to PCA and KPCA. In the Siberian data analysis, our method more clearly distinguishes the two populations even when limited to the genetic information from one chromosome.

Our method is particularly effective in increasing the power for low-coverage sequencing data, thus offering an option for researchers with a limited budget to study in medical and population genetics as well as assessing population structure for threatened or endangered species. With the advantage in low-coverage data, we believe MCPCA offers an attractive approach to the study of non-model organisms [7], which are often associated with the absence of closely related reference genomes and challenging sample material issues. The limitations of our method include, (1) MCPCA is likely to be computationally intensive if the number of SNPs used are large or the number of PCs output are large; (2) Although, discretization of the dosage values is deem necessary for MCPCA method, it might lead to loss of information. For these limitations, we defer to the future researches.

## Conclusions

In this paper, we introduce a dimension reduction tool MCPCA_PopGen to analyze population structure of low-depth sequencing data.

## Methods

### Find optimal MCPCs

Let $\mathbf{X}$ be a $n \times p$ matrix and its (*i*, *j*)th element be the discretized dosage value for the *i*th individual at the *j*th SNP. Let $\mathbf{x}^j \in \mathbb R^n$ represent a vector of dosage values of *j*th SNP across *n* individuals, and define the nonlinear transformations as $\phi =(\phi _1,\ldots ,\phi _p)$. Thus $\phi _1(\mathbf{x}^1), \cdots , \phi _p(\mathbf{x}^p) \in \mathbb R^n$ are the vectors of transformed dosage values. We restrict ourselves to standardized transformations and consider the collection of covariance matrices,

1 $$\begin{aligned} {\mathcal K}_{\mathbf{X}} =&\Big \{ {\mathbf{K}}_\phi \in \mathbb {R}^{p\times p}, {\mathbf{K}}_\phi (j,j') = E[\phi _j(\mathbf{x}^j)\phi _{j'}(\mathbf{x}^{j'})] : \nonumber \\&\qquad E[\phi _j(\mathbf{x}^j)]=0, E[\phi _j(\mathbf{x}^j)^2]=1\ \hbox {for}\ j,j'=1,\ldots ,p\Big \}. \end{aligned}$$

For a given value of *q*, [11] proposed the choice $\phi ^*=(\phi _1^*, \ldots ,\phi _p^*)$, ${\mathbf{K}}_{\phi ^*} \in {\mathcal K}_{\mathbf{X}}$, to maximize the sum of the top *q* eigenvalues, i.e., $\phi ^*$ achieves the Ky Fan *q*-norm

2 $$\begin{aligned} \phi ^*=\arg _{\phi } \max _{{\mathbf{K}}_\phi \in {\mathcal K}_{\mathbf{X}}}\sum _{r=1}^{q}\lambda _r({\mathbf{K}}_\phi ), \end{aligned}$$

where $\lambda _r({\mathbf{K}}_\phi )$ is the *r*th largest eigenvalue of ${\mathbf{K}}_\phi$. MCPCA thus can be considered as a generalization of PCA over all possible nonlinear transformations of predictors. The *q* optimal maximally correlated principle components (MCPCs) achieve the Ky Fan *q*-norm. Because PCA is based on computing eigenvalues for the special choice of $\phi$ where each component $\phi _j$ is a linear function, the sum of the top *q* eigenvalues for PCA is upper bounded by the Ky Fan *q*-norm.

To solve this optimization problem, we adopted the block coordinate descent algorithm [11]. Implementation of the algorithm to genetic data requires, as with PCA, replacing the expectations in (1) with sample means.

### Discretize dosage values

Discretization of the dosage values is necessary to create a computationally feasible algorithm. We have previously evaluated several discretization protocols. The equal width, equal frequency, and Jenks binning methods are considered [18], with the number of bins, *m*, determined by the Freedman-Diaconis rule (equation (S1) in Additional file 1). The discretization method is performed over each SNP individually. For equal width binning method, we divide the range of the dosage values for a given SNP into *m* bins, with each bin having equal interval length. For equal frequency binning method, we use a similar strategy by replacing the range of dosage values with their frequencies. Each category thus has an equal number of members. However, if the data contain duplicated values, the equal frequency binning may not achieve perfect equally sized groups. For Jenks binning, we partition the dosage values into *m* clusters such that the within-cluster variations are minimized and between-cluster variations are maximized. To avoid label switching problem in Jenks binning, we assign the labels to the *m* clusters according to their group means. We evaluated the performance of MCPCA using the equal width, equal frequency, and Jenks binning methods. For ease in presentation, we refer to discretization methods as MCPCA-Intv, MCPCA-Freq, and MCPCA-Jenks respectively.

### Simulation

We evaluate MCPCA_PopGen using three types of genotype callings.

- **Perfectly known genotypes.** To simulate the genotype data under a variety of assumptions concerning migration, recombination rate, and population size under neutral models, we used a coalescence simulator *ms* to simulate haplotypes for 50 individuals from each of three populations (African, Caucasian and Asian) [17]. Then we generated the genotypes of admixed individuals based on the *ms* output (See **Supplemental Material** for *ms* commands adopted to generate genotypes from admixed populations). After obtaining genotypes, we filtered out rare variants with minor allele frequency (MAF) below 0.05. These data play the role of perfectly known genotypes that come with high coverage NGS. The genotype $G_{ij}$ is treated as the minor allele counts (i.e., 0, 1, 2) carried by individual *i* at each locus *j*.
- **Observed genotypes (with error).** We generated the observed genotypes $\tilde{G}_{ij}$ under different coverage depths by perturbing $G_{ij}$ with sequencing qualities sampled from the 1000 Genomes project [8, 9]. More specifically, we simulated $\tilde{G}_{ij}$ by perturbing $G_{ij}$ using errors generated from the Bernoulli distribution with probability $\epsilon _{ij}=10^{-Q_{ij}/10}$, where $Q_{ij}$ is the quality score determined by the coverage depth. At a given mean depth, the number of reads for each genotype was sampled from *Gamma* distribution with shape and scale parameters 6.3 and *depth*/6.3 [8, 31, 38]. Then $Q_{ij}$ was sampled from the quality scores in the 1000 Genomes project whose observed number of reads is closest to the number of reads simulated from mean coverage. Thus, we generated the observed genotypes $\tilde{G}_{ij}$’s along with the corresponding base-calling error probabilities $\epsilon _{ij}$’s.
- **Dosage genotypes.** Dosage genotypes are the posterior mean of the genotype under additive coding. With values 0, 1 and 2 assigned to the genotypes (major, major), (major, minor) and (minor, minor), respectively, the dosage, ${\text{ DS }} = \Pr (1 |{\text{ Data}})$ + 2$\Pr (2 | {\text{ Data}})$, where $\Pr (1 |{\text{ Data}})$ and $\Pr (2 |{\text{ Data}})$ denote the conditional (“posterior”) probabilities for the genotypes (major, minor) and (minor, minor). Our method can also be applied to dosage data imputed by Mach/Thunder [23].

### Implementation

MCPCA_PopGen is an open-source package. The source code of MCPCA is provided by [11] using Matlab. To make it easier to install and implement, we provide the entire package MCPCA_PopGen in the high-performance Julia language. Both the *ms* commands for generating genotypes and the documented source code for MCPCA_PopGen are hosted on GitHub: https://github.com/yiwenstat/MCPCA_PopGen.

## Supplementary Information

**Additional file 1.** Details of estimating nonlinear transformation, discretization schemes, and simulation commands.

## Data Availability

The full exome data for the two Siberian population samples are publicly available from the NCBI Sequence Read Archive with accession BioProjectID: PRJNA389435. The package is available on GitHub.

## Abbreviations

NGS: Next-generation sequencing
DNA: Deoxyribonucleic acid
PCA: Principal component analysis
PC: Principal component
LLE: Locally linear embedding
KPCA: Kernel PCA
MCPCA: Maximally correlated principal component analysis
MCPC: Maximally correlated principal component
MCPCA_PopGen: Maximally correlated principal component analysis application to population structure with low-coverage sequencing data
MAF: Minor allele frequency
MCPCA-TG: MCPCA algorithm is applied to the true genotype data
MCPCA/PCA-DS: MCPCA/PCA algorithm is applied to the genotype dosage data
MCPCA-Intv: MCPCA algorithm applied to discretized dosage data binning methods by equal width
MCPCA-Freq: MCPCA algorithm applied to discretized dosage data binning methods by equal frequency
MCPCA-Jenks: MCPCA algorithm applied to discretized dosage data binning methods by Jenks binning
MCPCA/KPCA/PCA-OG: MCPCA/KPCA/PCA algorithm applied to observed genotypes
OOB: Out-of-bag
YAK: Yakut
SNP: Single nucleotide polymorphism
