# Supplementary material for: A novel nonlinear dimension reduction approach to infer population structure for low-coverage sequencing data
Source: BMC Bioinformatics. 2021 Jun 26;22:348. doi: 10.1186/s12859-021-04265-7 (PMC8236193; doi:10.1186/s12859-021-04265-7)
Supplement: Supplementary file 1 — Additional file 1. Details of estimating nonlinear transformation, discretization schemes, and simulation commands. [file 12859_2021_4265_MOESM1_ESM.pdf]

Supplementary Material for "A novel nonlinear dimension reduction approach to infer population structure for low-coverage sequencing data"

Miao Zhang [1†], Yiwen Liu [2†], Hua Zhou [3], and Joseph Watkins [1,2], Jin Zhou [1,4*]

[1]Interdisciplinary Program in Statistics and Data Science, University of Arizona, Tucson, 85721, USA,

[2]Department of Mathematics, University of Arizona, Tucson, 85721, USA,

[3]Department of Biostatistics, University of California, Los Angeles, 90095, USA,

[4]Department of Epidemiology and Biostatistics, University of Arizona, Tucson, 85724, USA.

[*]To whom correspondence should be addressed.

[†] Equal contributor.

1 Estimating nonlinear transformation

As illustrated in Figure S1, the distribution of dosage genotypes is closer to that of true genotypes under coverage 10× and deviates from the distribution of true genotypes under extremely low coverage, illustrated using 1× coverage. It is challenging to capture the low-frequency alleles under such low coverage with many low-frequency alleles treated as common variants. In order to visualize how the MCPCA algorithm chooses the nonlinear transformation ϕ, we bin loci into three groups according to MAF, low (MAF between 0 and 0.1), medium (MAF between 0.1 and 0.4), and high (MAF between 0.4 and 0.5). Figure S1 shows a local polynomial regression of the transformations at 5× coverage. For low MAF, the transformed dosage emphasizes the uncertainty in calls between heterozygous and major homozygous loci. For high MAF, the transformation is nearly linear.

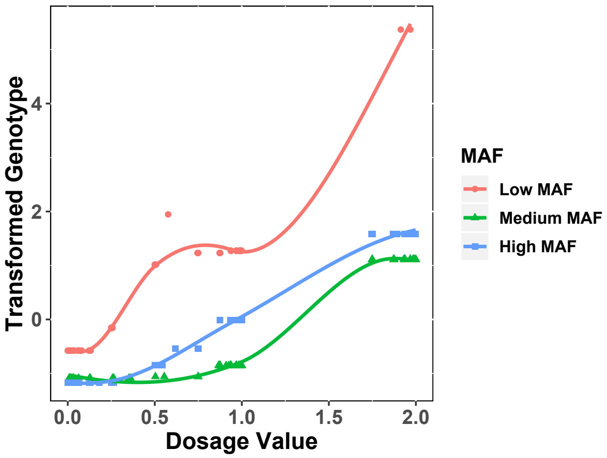

Figure S1: The relationship between transformed genotype and the dosage value. Loci with low (MAF<0.1), medium ($0.1 \leq$MAF< 0.4), and high MAF (MAF≥ 0.4) were selected. The horizontal axis represents dosage values, and the vertical axis is the transformed genotype using MCPCA. All the lines are fitted by local polynomial regression. The data were generated using *ms* simulator with coverage depth 5.

2 Determine the discretization scheme for dosage values

The MCPCA algorithm requires discretization of the dosage. To counteract both overfitting and underfitting, we consider data-driven approaches. The number of bins to discretize genotype probability distribution is determined by minimizing the L_2 distance between the actual density and the histogram. Restricting the consideration to equal width bins, a good approximation of the bin width, namely $2\,\mathrm{IQR}/\sqrt[3]{n}$, serves this goal (Freedman and Diaconis, 1981). Here IQR is the interquartile range, and n is the number of observations.

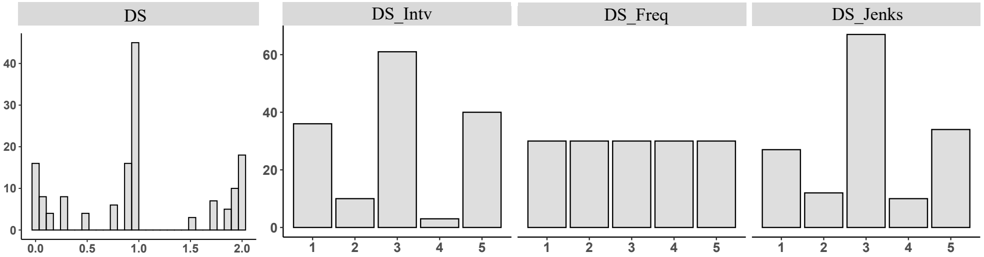

Figure S2: An example of the histogram of dosage values (left) and discrete dosage values with 3 discretization methods: equal interval, equal frequency, and Jenks method (right).

With dosage values in the interval $[0, 2]$, this results in

$$\frac{\sqrt[3]{n}}{\text{IQR}} \tag{S1}$$

bins. Under low coverage depth, we observe more dosage genotypes cluster around 0.5 and 1.5 (Figure 2). With lower IQR, more categories are assigned based on equation (S1) to handle the uncertainty in dosage data with lower coverage depth.

In addition to equal width bins, we also consider equal frequency and Jenks optimization binning methods (Jenks, 1967) keeping the number of bins in equation (S1). The dosage values under low coverage ($5\times$), not surprisingly, have most values clustered around the values 0, 1 and 2 (Figure S2 left panel). Compared to equal width and Jenks binning, equal frequency binning more strongly equalizes the numbers of observations for each category (Figure S2).

3 Simulated data

The code we used to generate the data is:

> *ms* 1500 1 -t 935.68 -I 3 500 500 500 -r 935.68 1000 -n 1 1.980002 -n 2 4.914199 -n 3 6.696391 -eg 0 2 110.450397 -eg 0 3 139.447680 -ma x 0.743706 0.227223 0.743706 x 0.911111 0.227223 0.911111 x -ej 0.032140 3 2 -en 0.032140 2 0.254609 -ema 0.032140 3 x 4.457636 x 4.457636 x x x x x -ej 0.070325 2 1 -en 0.202422 1 1.

We have consulted with a population genetics professional for all the parameter settings.

References

Freedman, D. and Diaconis, P. (1981). On the histogram as a density estimator:l 2 theory. *Zeitschrift for Wahrscheinlichkeitstheorie und Verwandte Gebiete*, 57(4):453–476.

Jenks, G. F. (1967). The data model concept in statistical mapping. *International yearbook of cartography*, 7:186–190.
